# Increased Stem Cell Marker Expressions during the Peri-Implantation Period in the Rat Endometrium: Constructive Role of Exogenous Zinc and/or Progesterone

**DOI:** 10.1155/2014/867131

**Published:** 2014-05-26

**Authors:** Cagdas Sahin, Ozlem Yilmaz Dilsiz, Sirin Bakti Demiray, Ozgur Yeniel, Mete Ergenoglu, Ebru Demirel Sezer, Gulperi Oktem, Ege Nazan Tavmergen Goker, Erol Tavmergen

**Affiliations:** ^1^Department of Obstetrics and Gynecology, Tepecik Training and Research Hospital, 35170 Izmir, Turkey; ^2^Department of Histology and Embryology, Medicine Faculty, Ege University, 35040 Izmir, Turkey; ^3^Department of Obstetrics and Gynecology, Medicine Faculty, Ege University, 35040 Izmir, Turkey; ^4^Department of Biochemistry, Medicine Faculty, Ege University, 35040 Izmir, Turkey

## Abstract

*Background*. The aim of this study is to determine the effects of zinc and/or progesterone via the expression of **α**v**β**5 integrins and Vitronectins and embryonic stem cell markers during the peri-implantation period. *Methods*. Four experimental groups were organized. All subjects were mated with males of the same strain to induce pregnancy; after 5 days, zinc and/or progesterone were administered. Blood levels of zinc and progesterone were determined on the sixth day and endometrial tissues were obtained in order to evaluate the immunohistochemical expression of integrins and embryonic stem cell markers. *Results*. The **α**v**β**5 integrin and vitronectin expression increased in the zinc group compared with the control group and no difference in the progesterone group and zinc + progesterone group. Expression of Klf-4, Sox-2, and c-Myc was found to be increased in the zinc group compared to controls, while no difference was determined between the progesterone, zinc + progesterone, and control groups. Distinctively, expression of the embryonic stem cell marker Oct-4 was increased in all of the experimental groups. *Conclusions*. Expression of **α**v**β**5 integrin, vitronectin, and embryonic stem cell markers might be increased by the administration of zinc. Our results suggest that zinc could be useful in the induction of implantation rates.

## 1. Introduction

Today, infertility is an important health problem due to the increased age of those individuals wishing to have children. The incidence of infertility is 10–15% in those of reproductive age, and this proportion is steadily rising [1].

Implantation occurs at 5 or 6 days after fertilization. Integrins (e.g., *α*v*β*3 and vitronectin) have an important role in the embryo-endometrial interaction at the time of implantation [2, 3]. The *α*v*β*5 integrin has a similar function to *α*v*β*3 integrin [4] and vitronectin is the stromal receptor of the *α*v*β*3 integrin [5]. We studied these integrins as there are few studies in the literature about the role of these integrins in reproduction.

On the other hand, the human endometrium has remarkable regenerative capacity due to the presence of endometrial stem cells [6]. However, there is a gap of knowledge about the role of these endometrial stem cells in the implantation period. When the role of stem cells in implantation is revealed, infertility of unknown causes may also be explained; therefore, this study was considered a step towards this knowledge.

Progesterone is essential for the development of uterine receptivity through activity in both the epithelial and stromal compartments and dysfunction of progesterone response may be a hallmark of a variety of conditions associated with infertility and endometrial pathology. Progesterone acts indirectly through the stromal cells and might increase growth factor production in stromal cells, as it is a paracrine regulator of epithelial gene expression [7]. A more direct (endocrine) role of progesterone is performed via the direct stimulation of gene expression in epithelial cells [7].

Zinc plays a role in DNA transcription and as a cofactor of metalloenzymes which have a role in protein synthesis and also as a cofactor of zinc finger transcription factor, which is a key component of the network, it is required to maintain pluripotency [8, 9]. Zinc is located in the center of many important processes in reproduction [9].

The aim of this study is to evaluate the effects of zinc and/or progesterone on the expression of *α*v*β*5 integrins and vitronectin in endometrial embryonic stem cells in the peri-implantation period. As a result, the issue of infertility due to the failure of implantation will hopefully be resolved.

## 2. Materials and Methods

The study protocol complied with the European Community Guidelines for the Use of Experimental Animals. All experiments were approved by the Local Animal Care Ethics Committee at Ege University (2010-10).

### 2.1. Animals

Virgin, 8–12-week-old female Wistar Albino rats (200–220 g) were housed in the animal care facility with a constant 12-hour light/dark cycle and fed* ad libitum*. Cages were kept at 18–21°C with relative humidity at 45–75%.

### 2.2. Determination Cycle of Rats

Four stages of the regular 4-day estrous cycle of the rat were determined by examining vaginal smears according to the criteria described by Rogers and Gannon [10]. The rats in the same cycle were housed in the same cage. We organized 4 groups as follows: *n* = 11 for the control group, *n* = 10 for the progesterone group, *n* = 9 for the zinc group, and *n* = 13 for the zinc + progesterone group.

### 2.3. Pregnancy

All subjects were mated with males of the same strain to induce pregnancy. Determination of a vaginal plug on the following date was considered indicative of pregnancy and this time point was considered day 1 of pregnancy.

### 2.4. Zinc and Progesterone Application

Zinc and/or progesterone were applied during the first five days of pregnancy. Zinc sulfate heptahydrate (Z0635; Sigma-Aldrich) was applied at a dose of 2 mg/kg intraperitoneally for 5 days. Progesterone (progynex 50 mg/mL, Farmako) was applied at a dose of 1 mg/rat by intramuscular injections for 5 days. The control group received neither of the drugs.

### 2.5. Tissue Processing

Removed uteri were fixed by overnight immersion in 4% paraformaldehyde (Merck & Co., Inc., USA); then, samples were dehydrated in ascending alcohol solutions, embedded in paraffin, and sectioned via a microtome (Leica RM 2145). For immunohistochemical analyses, 5 *μ*m thick sections were used for the following primary antibodies: Sox2 (Abnova, Taipei, Taiwan), c-Myc (Santa Cruz Biotechnology, Santa Cruz, CA), Oct4, and Klf4 (Abcam, Cambridge, MA), all of which were diluted at 1/300. Both investigators, blinded to the group distinctions of the specimens, obtained five images from 10 different sections under ×100 magnification. The intensity of Klf4, Sox2, c-Myc, and Oct4 immunohistochemical staining was graded semiquantitatively according to the nuclear, cytoplasmic, or extracellular immunoreaction in uterine sections as follows: (−) no immunostaining, (+) weak staining, (++) moderate staining, and (+++) strong staining.

### 2.6. Biochemical Analysis

The plasma progesterone assay was carried out with the Progesterone Enzyme Immunoassay Kit (Assay Design Inc.; Ann Arbor, MI, USA). Plasma zinc levels were measured with the colorimetric QuantiChrom Zinc Assay Kit (DIZN-250, Bioassay Systems, CA, USA) where a chromogen formed a colored complex specifically with zinc; the intensity of the color was measured at 425 nm.

## 3. Results

The intensity of immunoreactivity was graded semiquantitatively; scores are shown in Table 1.

### 3.1. Enhanced Expression of *α*v*β*5 Integrin following Zinc and/or Progesterone Administration

**Figure 1:**
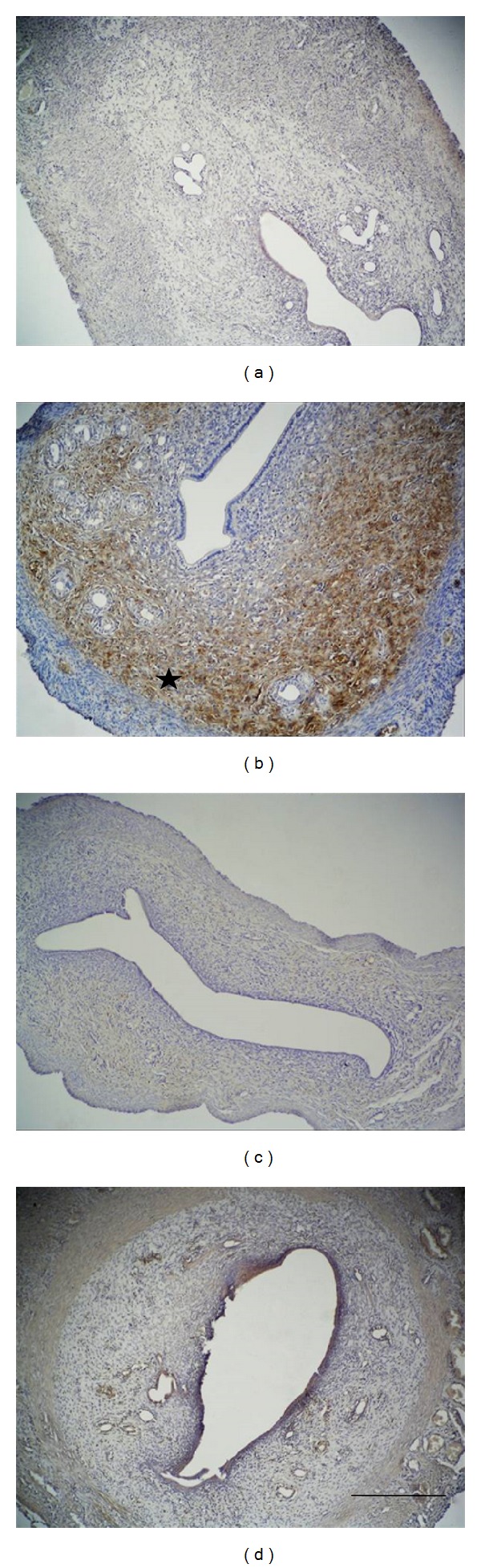
Observed *α*v*β*5 integrin immunoreactivity (starred) in the extracellular matrix has increased especially in the zinc group in comparison to the control group: control group (a), zinc group (b), progesterone (c), and zinc + progesterone combination group (d). Scale bar is 125 *μ*m; original magnification is 10x.

Compared with the control group, prominent findings of increased expression were especially determined in the basal layer of functional endometrial glands and in the surrounding stromal localized areas of the zinc group, zinc + progesterone group, and progesterone group in particular. There was no expression of endometrial surface epithelium and glandular epithelium in any of the groups (Figure 1).

### 3.2. Enhanced Expression of Vitronectin following Zinc Administration

**Figure 2:**
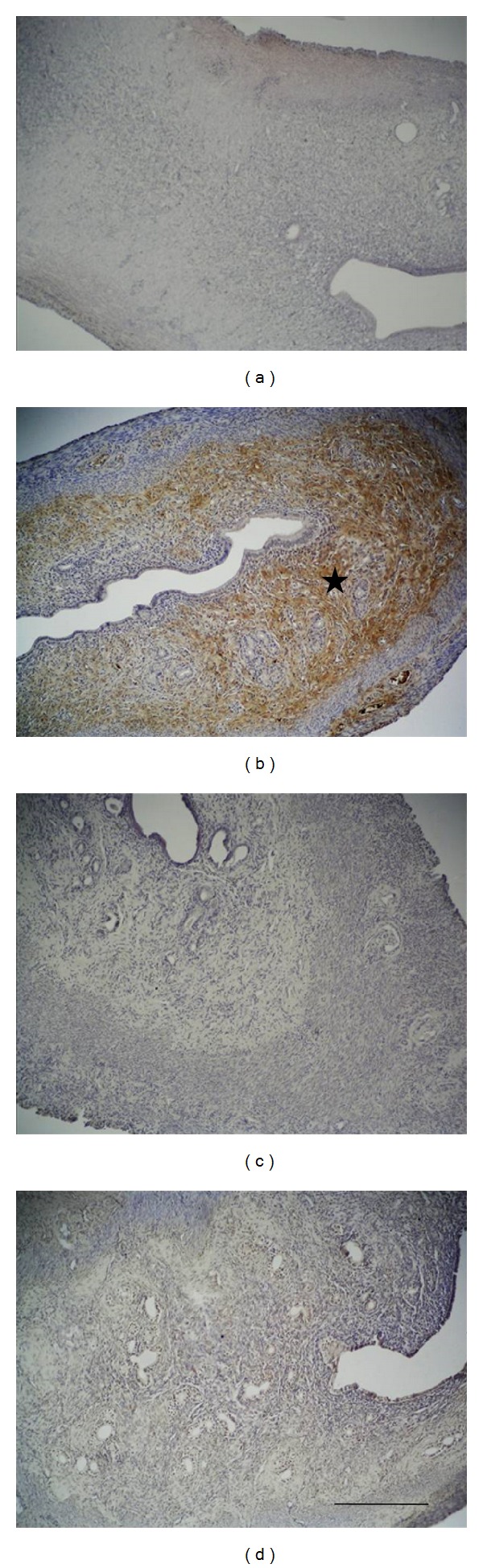
Vitronectin immunoreactivity in the extracellular matrix (starred) in the zinc group has increased significantly compared to the control group. Progesterone and zinc + progesterone group showed similar immunoreactivity to the control group: control group (a), zinc group (b), progesterone (c), and zinc + progesterone group (d). Scale bar is 125 *μ*m; original magnification is 10x.

Among all of the experimental groups, immunoreactivity of vitronectin revealed findings of increased expression especially for the zinc group in the superficial stroma and glandular epithelium, as well as in the basal layer of the endometrium, while the controls and other experimental groups did not show any difference in the basal levels of immunoexpression (Figure 2).

### 3.3. Enhanced Expression of Klf-4 following Zinc Administration

**Figure 3:**
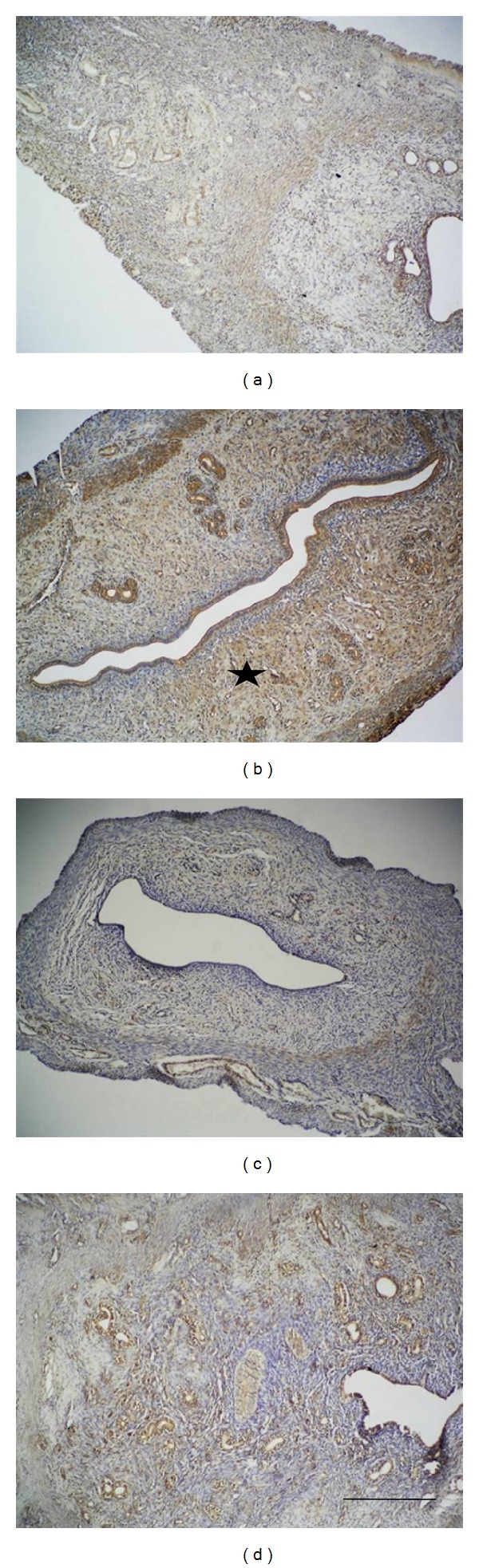
Compared to the control group, Klf-4 immunoreactivity (starred) in the zinc group was significantly increased. Immunoreactivity of progesterone and zinc + progesterone group revealed similar results to the control group: control group (a), zinc group (b), progesterone (c), and zinc + progesterone group (d). Scale bar is 125 *μ*m; original magnification is 10x.

Compared to the control group, Klf-4 immunoreactivity in the zinc group was significantly increased. Increased expression for the zinc group was localized in particular in the basal region of the endometrium and the epithelium of the endometrial glands significantly. Compared to the control groups, the basal expression profiles of the immunoreactivity of Klf-4 for the progesterone and zinc + progesterone groups did not show any significant differences (Figure 3).

### 3.4. Enhanced Expression of Sox-2 following Zinc Administration

**Figure 4:**
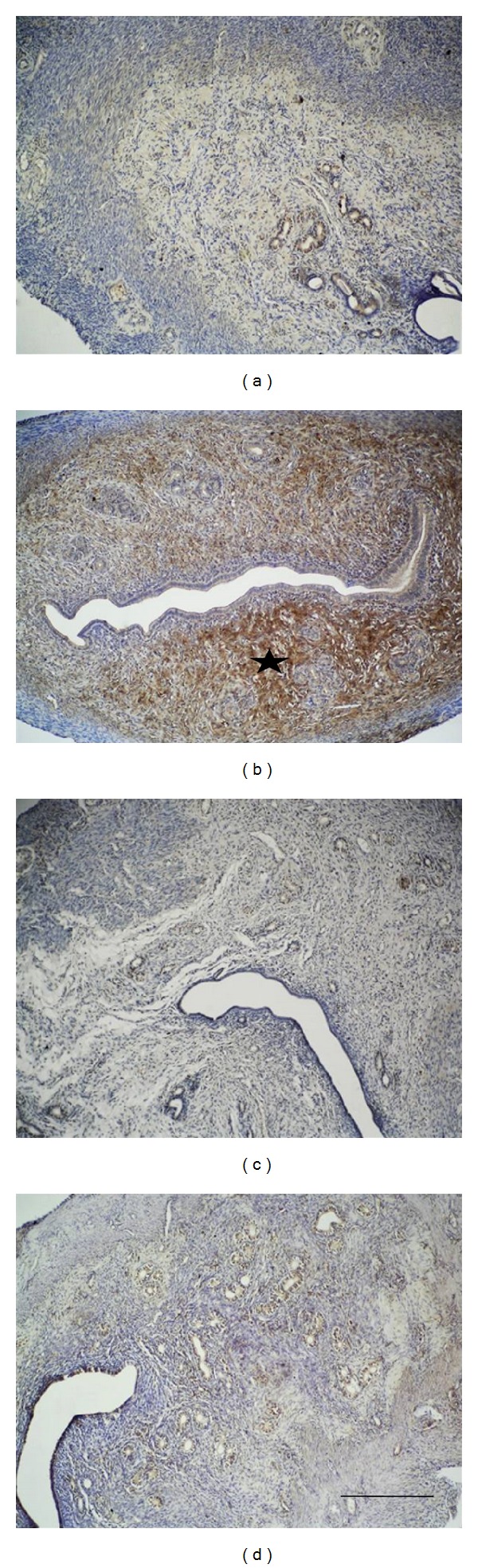
Sox-2 immunoreactivity (starred) in the zinc group was significantly increased compared to the control group. Progesterone and zinc + progesterone group revealed similar immunoreactivity to the control group: control group (a), zinc group (b), progesterone (c), and zinc + progesterone group (d). Scale bar is 125 *μ*m; original magnification is 10x.

Sox-2 immunoreactivity in the zinc group was significantly increased compared to the control group. The progesterone and zinc + progesterone groups revealed similar immunoreactivity to the controls. In the control group, a significant positive reaction was determined, especially in the glandular tissue. The progesterone and zinc + progesterone groups revealed immunoreactivity profiles that were similar to the controls. Findings of significantly increased expression were reported for the zinc group, which were different to the 3 specified groups, especially in the glandular epithelium, stroma, and basal layer of the endometrium (Figure 4).

### 3.5. Enhanced Expression of c-Myc following Zinc Administration

**Figure 5:**
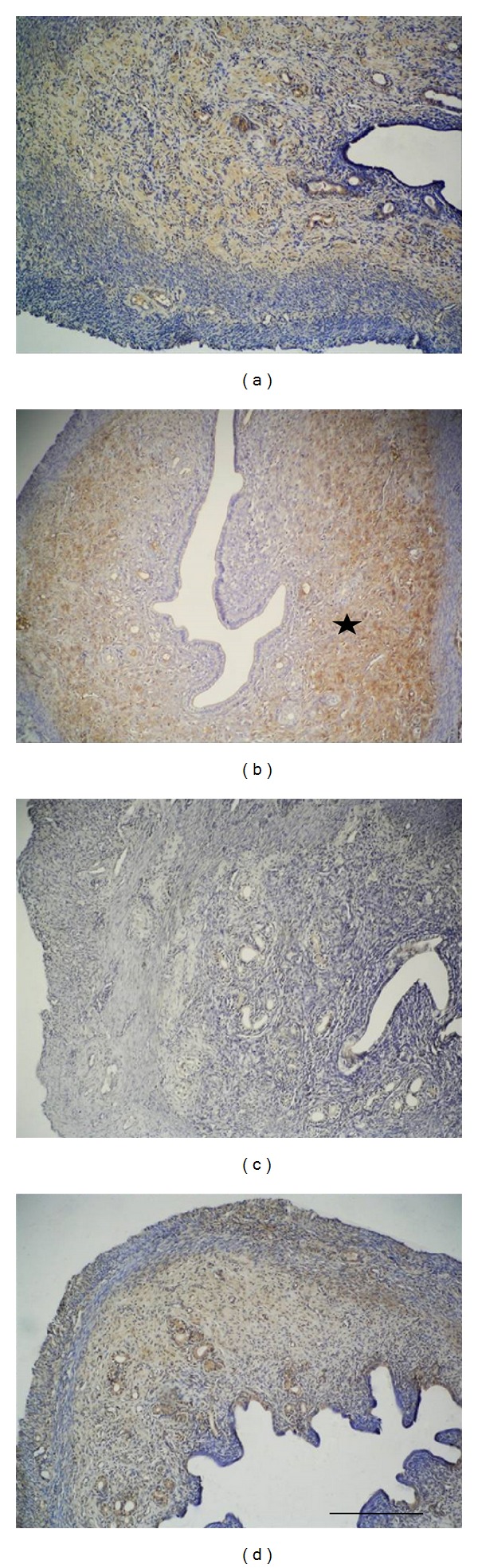
c-Myc immunoreactivity in the zinc group (starred) revealed significant increase compared to the control group. Progesterone and zinc + progesterone group showed similar immunoreactivity to the control group: control group (a), zinc group (b), progesterone (c), and zinc + progesterone group (d). Scale bar is 125 *μ*m; original magnification is 10x.

c-Myc immunoreactivity in the zinc group was significantly increased compared to the control group. The progesterone and zinc + progesterone groups showed similar immunoreactivity to the controls. Evidence of basal expression levels was partially observed for the control, progesterone, and zinc + progesterone groups in the endometrial glandular epithelia and the stroma showed increased expression, particularly for the zinc group. The immunoreactivity of the c-Myc profile was apparent, especially in the basal layer of the endometrium for the zinc group (Figure 5).

### 3.6. Enhanced Expression of Oct-4 following Zinc and/or Progesterone Administration

**Figure 6:**
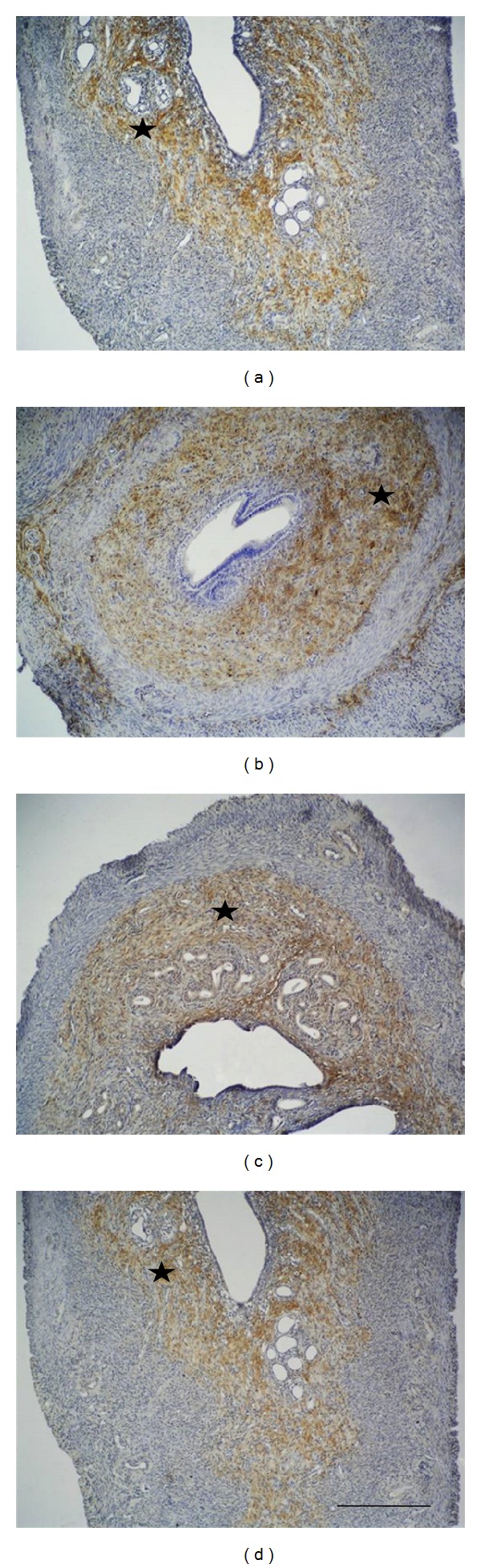
Oct-4 immunoreactivity in the zinc group, progesterone group, and zinc + progesterone group revealed a slight increase in comparison to the control group (starred): control group (a), zinc group (b), progesterone (c), and zinc + progesterone group (d). Scale bar is 125 *μ*m; original magnification is 10x.

Oct-4 immunoreactivity in the zinc, progesterone, and zinc + progesterone groups revealed a slight increase in comparison to the control group. In the endometrial stroma, there was increased expression of Oct-4 in the 3 study groups, whereas this was mild for the control group. The increased expression in the progesterone and zinc + progesterone groups was localized particularly in the endometrial surface epithelium while no such expression was determined for the control and zinc groups. Increased glandular immunoreactivity of Oct-4 was apparent for the progesterone group (Figure 6).

### 3.7. Biochemical Results

#### 3.7.1. Serum Zinc Level

The serum zinc level in the zinc and the zinc + progesterone groups was determined to be higher in cases that reached a certain serum zinc level compared to the control groups (Table 2).

#### 3.7.2. Serum Progesterone Level

Serum progesterone level in the progesterone and the zinc + progesterone groups was higher in those that reached a certain serum progesterone level compared to the controls (Table 3).

## 4. Discussion

Implantation is one of the most important steps in pregnancy. The first and most important step of the implantation stage is adhesion, which is the basis of all other stages. Adhesion molecules, especially the integrin family of proteins, have important roles in this stage. Previous literature has highlighted the expression of 3 integrin molecules during the peri-implantation period [11].

Vitronectin is located in the extracellular matrix (ECM) of the endometrium. Several integrins easily recognize vitronectin, such as *α*v*β*3, the platelet gpIIbIIIa, the “fibrinogen receptor,” and other *α*v integrins, including *α*v*β*5 and *α*v*β*l. Osteopontin, which is another extracellular matrix protein, is the ligand of *α*v*β*5 integrin and previous studies have revealed its regulation via progesterone [12]; however, there have been no such studies about vitronectin. This study showed that vitronectin expression was similar for both the progesterone and control groups. Exogenous progesterone had no effect over the basal expression of vitronectin, while vitronectin expression was increased in the zinc group in comparison to the controls. Thus, zinc potentiated the therapeutic effects of vitronectin. The zinc + progesterone group showed similar expression patterns to the control group, which revealed that progesterone repressed the zinc effect. This situation was confusing, however. Zinc finger proteins are involved in the expression of steroid hormone receptors [8, 9] and zinc supplementation has already proven to be beneficial in male sterility and in reducing complications during pregnancy, as shown by using steroid receptors which are comprised of zinc finger proteins [13]. Thus, we think that the results of this study demonstrate that exogenous progesterone induces the production of receptors and exogenous zinc administration has an effect on zinc finger protein function. There is clear competition between zinc and progesterone.

The determined increase in serum progesterone levels showed that epithelial *α*v*β*3 integrin expression occurred due to the role of progesterone [3]. The integrin *α*v*β*5 has been demonstrated to show expression patterns similar to *α*v*β*3 integrins on endometrial epithelium, stroma, and embryo surfaces [4]. As a result, *α*v*β*5 integrin may have similar functions during the implantation period. Previous results in the literature have shown that the *α*v*β*5 integrin is present in the luminal epithelium and stroma in both the human and mouse endometrium at the time of implantation [2]. The results of this study also revealed that *α*v*β*5 expression was similar in both progesterone and control groups, while it was increased in the zinc group. These findings show the increased effects of zinc on the expression patterns of *α*v*β*5 integrins; however, interestingly, progesterone did not affect the expression of integrins. In the zinc + progesterone combination group, the expression of *α*v*β*5 was similar to that seen in the control group, where progesterone decreased the effects of zinc function. Therefore, the results of this study support our theory regarding the competition between zinc and progesterone in zinc finger proteins.

Steroid hormones play a role in integrin regulation in the secretory period of the cycle [3, 14], but our study results showed that exogenous progesterone has no additional contribution in the regulation of vitronectin and the *α*v*β*5 integrin. Yet, here, the results of this study revealed a novel effect of zinc on integrin expression, which has been reported immediately after implantation for the first time in the literature.

Zinc is an essential nutrient for fetal development and optimal fertility and previous studies have shown that chronic maternal zinc deficiency leads to abnormal fetal development, birth defects, low birth weight, and severely disrupted oocyte maturation, fertilization, and preimplantation development in numerous species, including humans [15–18]. Whilst zinc is known to be less toxic than other metals such as mercury, lead, arsenic, and cadmium, acute and chronic toxicities after excessive exposure have been reported [19].

In a zinc toxicity study, it was shown that more than 80% of malformations occurred at an exposure level of 1.0 mg/L in* Gobiocypris rarus* embryos [20] and the value for the oral lethal dose 50 (LD 50) of zinc was found to be 2000 mg/kg for female rats [21]. Another study has shown mild adverse toxic effects on reproductive endpoints and liver function in adult rats, as well as abnormal developmental effects in offspring when supplemented with Zn at concentrations of 7.5, 15, and 30 mg/kg [22]. However, zinc toxicity in humans is considered rare, but it has been known to occur [23].

These results indicate that excess zinc supplementation before and during pregnancy as well as during lactation could pose some health risk concerns to pregnant mothers and their offspring. In previous studies it was used at low doses (<2000 mg/kg), where no toxicity or side effects were reported [21]. We used very low doses of zinc (1 mg/kg) and did not observe any toxic effects.

Recently, it has been shown that adult stem cells are undifferentiated cells present in many adult tissues and organs. These cells show characteristics like self-renewal and differentiation into one or more lineages and hold high proliferative potential [24]. Adult stem cells maintain tissue homeostasis despite the provision of replacement cells in both routine cellular turnover and also the repair of injured tissues [25]. Adult stem or progenitor cells are responsible for the cyclic regeneration of the functional layer of the endometrium each month, and these adult stem cells reside in the basal layer and are even present in the atrophic endometrium of postmenopausal women [6]. Thus, under estrogen replacement therapy, postmenopausal endometrial regeneration occurs through an adult stem cell population located in the basal endometrium [6, 26].

Embryonic stem cell markers include Sox-2, Oct-4, Klf-4, and c-Myc, and human endometrial cells yield significantly higher numbers of embryonic stem cell markers in comparison to neonatal skin fibroblasts [26]. Oct4 [27] and Sox2 [28] function in the maintenance of pluripotency of both early embryos and embryonic stem cells. On the other hand, both Klf4 and c-Myc are dispensable for preimplantation mouse development [29, 30], and their role in implantation is unknown. Oct-4 is not differentially expressed during the menstrual cycle [29]. Our hypothesis is that zinc administration increased Oct-4 expression in the peri-implantation period, potentially to facilitate embryo implantation.

A number of studies have demonstrated that local injury to the endometrium by endometrial biopsy increases implantation and pregnancy rates in subsequent IVF-embryo transfer cycles [31–33]. This injury may also recruit stem cells to the endometrium; adult stem cells have been shown to give rise to endometrium, perhaps creating a partially new endometrium that is free of epigenetic defects [34, 35]. Thus, if the endometrial adult stem cell population is increased by zinc administration, this may trigger the increase in implantation and pregnancy rates.

Zinc finger proteins play an important role in the regulation of pluripotent and embryonic stem cells. Studies have shown the role of zinc finger protein 281 [36] and zinc finger protein 206 [37] in the regulation of embryonic stem cells [37]. Regulation of these proteins may affect the turnover of embryonic stem cell life cycles. In our study, the zinc group showed increased expression of the embryonic stem cell markers compared to other groups, which led us to consider that zinc finger proteins contribute to this expression. As already mentioned, zinc finger proteins are involved in the genetic expression of progesterone receptors [8, 38]. Exogenous progesterone activates zinc finger proteins, and active zinc finger proteins decrease the effect of zinc in tissue expression after the administration of exogenous zinc.

Progesterone is essentially required for the formation and continuation of pregnancy [12, 39, 40]. Exogenous progesterone administration did not increase the expression of embryonic stem cell markers, with the exception of Oct-4, compared to the control group. The administration of either zinc or progesterone increased the expression of Oct-4, but the combined zinc and progesterone administration did not show any pharmacological additive effects, probably due to the fact that both zinc and progesterone act through the same zinc finger proteins. It is our conclusion in this study that zinc is not sufficient for additive effects. To overcome this problem, the zinc levels may be increased.

In conclusion, the results of this study showed that exogenous progesterone administration did not affect the expression of *α*v*β*5 integrin, vitronectin, Klf-4, Sox-2, or c-Myc. However, this fact is not sufficient to diminish the conventional importance of progesterone during implantation.

As another important result of this study, zinc was shown to be a promising element for implantation. In particular, the effect of zinc on stem cells is of great value and needs to be explored in further detail with novel molecular techniques. Also, quantification of gene expression by confocal immunomicroscopy would provide an even better perspective to the subject. The semiquantitative evaluation of immunoreactivity was a limitation of the current study. However, this study demonstrated that Klf4, Sox2, Oct4, and c-Myc immunopositive cells in the adult endometrium manifested sustainable pluripotency. Although the reported alterations could be the consequence of endometrial turnover during the usual menstrual cycle, there is still a need for further sophisticated studies with other animal models of implantation such as the NOD or the Akita mouse to test this possibility.

## Figures and Tables

**Table 1:** Endometrial expression status of *α*v*β*v integrin, vitronectin, Klf4, Sox2, Oct4, and c-Myc.

Markers	Groups			
	Control group (*n* = 11)	Zinc group (*n* = 9)	Progesterone group (*n* = 10)	Zinc + progesterone group (*n* = 13)
*α*v*β*5 integrin	+	++	+	+
Vitronectin	+	+++	+	+
Klf-4	++	+++	++	++
Sox-2	+	+++	+	+
c-Myc	+	+++	+	+
Oct-4	++	+++	+++	+++

Median expression of each group and used Kruskal-Wallis test.

**Table 2:** Comparison of blood levels for zinc concentration in between the groups.

Groups	Subject number (*n*)	Zn^+2^ (*μ*g/mL)			*P*
		Mean	Median	Min.–max. values	
Control group	11	1.84	1.89	1.34–2.28	<0.005
Zinc group	9	2.20	2.34	1.82–2.54	<0.005
Progesterone group	10	1.77	1.79	1.37–2.06	<0.005
Zinc + progesterone group	13	2.01	2.05	1.53–2.60	<0.005

**Table 3:** Comparison of blood levels for progesterone levels in between the groups.

Groups	Subject number (*n*)	Progesterone (ng/mL)			*P*
		Mean	Median	Min.–max. values	
Control group	11	11.14	10.42	7.32–21.04	<0.005
Zinc group	9	10.81	10.54	7.88–14.02	<0.005
Progesterone group	10	151.17	152.15	96.45–220.42	<0.005
Zinc + progesterone group	13	134.75	123.32	76.90–210.60	<0.005
